# Combined transcriptome and metabolome analysis reveal key regulatory genes and pathways of feed conversion efficiency of oriental river prawn *Macrobrachium nipponense*

**DOI:** 10.1186/s12864-023-09317-1

**Published:** 2023-05-19

**Authors:** Feiyue Ling, Yaoran Fan, Zefei Wang, Nan Xie, Jiale Li, Guiling Wang, Jianbin Feng

**Affiliations:** 1 Key Laboratory of Freshwater Aquatic Genetic Resources, Ministry of Agriculture and Rural Affairs, Shanghai Ocean University, Shanghai, 201306 China; 2 National Demonstration Center for Experimental Fisheries Science Education, Shanghai Ocean University, Shanghai, 201306 China; 3 Hangzhou Academy of Agricultural Sciences, Hangzhou, 310012 China

**Keywords:** *Macrobrachium nipponense*, Feed conversion efficiency, Transcriptome; Metabolome, Key gene pathways

## Abstract

**Background:**

Oriental river prawn *Macrobrachium nipponense* is an economically important aquaculture species in China, Japan, and Vietnam. In commercial prawn farming, feed cost constitutes about 50 to 65% of the actual variable cost. Improving feed conversion efficiency in prawn culture will not only increase economic benefit, but also save food and protect the environment. The common indicators used for feed conversion efficiency include feed conversion ratio (FCR), feed efficiency ratio (FER), and residual feed intake (RFI). Among these, RFI is much more suitable than FCR and FER during the genetic improvement of feed conversion efficiency for aquaculture species.

**Results:**

In this study, the transcriptome and metabolome of hepatopancreas and muscle of *M. nipponense* from high RFI low RFI groups, which identified after culture for 75 days, were characterized using combined transcriptomic and metabolomic analysis. A total of 4540 differentially expressed genes (DEGs) in hepatopancreas, and 3894 DEGs in muscle were identified, respectively. The DEGs in hepatopancreas were mainly enriched in KEGG pathways including the metabolism of xenobiotics by cytochrome P450 (down-regulated), fat digestion and absorption (down-regulated) and aminoacyl-tRNA biosynthesis (up-regulated), etc. The DEGs in muscle were mainly enriched in KEGG pathways including the protein digestion and absorption (down-regulated), glycolysis/gluconeogenesis (down-regulated), and glutathione metabolism (up-regulated), etc. At the transcriptome level, the RFI of *M. nipponense* was mainly controlled in biological pathways such as the high immune expression and the reduction of nutrients absorption capacity. A total of 445 and 247 differently expressed metabolites (DEMs) were identified in the hepatopancreas and muscle, respectively. At the metabolome level, the RFI of *M. nipponense* was affected considerably by amino acid and lipid metabolism.

**Conclusions:**

*M. nipponense* from higher and lower RFI groups have various physiological and metabolic capability processes. The down-regulated genes, such as carboxypeptidase A1, 6-phosphofructokinase, long-chain-acyl-CoA dehydrogenase, et. al., in digestion and absorption of nutrients, and the up-regulated metabolites, such as aspirin, lysine, et. al., in response to immunity could be potential candidate factors contributed to RFI variation for *M. nipponense*. Overall, these results would provide new insights into the molecular mechanism of feed conversion efficiency and assist in selective breeding to improve feed conversion efficiency in *M. nipponense*.

**Supplementary Information:**

The online version contains supplementary material available at 10.1186/s12864-023-09317-1.

## Background

In commercial prawn aquaculture system, the fed diet principally depends on artificial formula feed made of grain and fish meal. In general, the feed cost usually accounts for a large part of the total input cost. Improvement of feed conversion efficiency would reduce feed cost, while simultaneously increase the production of edible food without increasing input factors. The feed conversion efficiency is an important trait for aquaculture species, and could be optimized by genetic breeding approach. The common indicators used for feed conversion efficiency include feed conversion ratio (FCR), feed efficiency ratio (FER), and residual feed intake (RFI) [1]. The FCR is the ratio of feed intake converted to the desired product output, the FER means the ratio between weight gain and feed consumption, and actually they are reciprocal in value with the same essences. The RFI implies the difference between predicted intake and actual feed intake based on the body weight, composition, and weight gain [2]. During the genetic improvement of feed conversion efficiency, a negative correlation existing between FCR and average daily gain (ADG) would induce fast-growing individuals with the lower FCR, and reduce the selection effect of them [3]. FCR and ADG were usually selected at the same time. However, RFI displays the phenotypically independent of growth performance [4], and the lower the RFI value, the higher the feed conversion efficiency. Compared to animals with the high RFI, the animals with the low RFI will consume less dry matter while maintaining similar growth performance. For most aquaculture species, there were growth performance dimorphism between male and female individuals. The further study has shown that there were significant differences in the FCR and no significant difference in the RFI between different sexes of Nile tilapia (*Oreochromis niloticus*) [5]. Therefore, using RFI as an indicator of feed conversion efficiency is more advantageous than FCR and FER.

Recent studies have shown that RFI has complex interactions with DNA transcription, RNA translation, and protein modification, and it is closely related to many metabolic pathways in organisms [6]. RFI has been estimated by protein turnover, tissue metabolism, and other factors such as digestibility and body composition in terrestrially farmed animals [7], such as cattle [8], sheep [9], pigs [10], chickens [11], etc., based on transcriptome and genomics. The RFI of fish such as rainbow trout (*Oncorhynchus mykiss*) showed an apparent genetic variation, and the individuals with lower RFI had a higher nitrogen retention rate and growth rate [12]. As we know, transcriptome and metabolome are powerful tools to characterize the genome-wide changes in transcripts and metabolites related the stress in aquaculture species, and combination of transcriptome and metabolome analysis could help to better understand the molecular mechanism of responses that affects phenotype in animals, so as to make breeding work more accurate and efficient [13].

Oriental river prawn *Macrobrachium nipponense* is an economically important aquaculture species in China, Japan, and Vietnam [14]. In commercial prawn farming, feed cost constitutes about 50 to 65% of the actual variable cost. Over the years, an enormous amount of research has been conducted to determine the nutritional requirements and feed formulation of this species [15]. To the best of our knowledge, no study has focused on the feed conversion efficiency of this prawn. The hepatopancreas is an essential organ for digestion and nutrient absorption [16], and muscle is a fundamental part of the metabolism and energy storage in *M. nipponense* [17]. In this study, the RFI profile of *M. nipponense* was characterized, and the highest and lowest RFI groups were identified after culture for 75 days feeding with the same feed. Furtherly, differential expression genes (DEGs) and differently expressed metabolites (DEMs) from the hepatopancreas and muscle of the highest and lowest RFI groups were identified by transcriptome and metabolome analysis, respectively. Finally, transcriptome and metabolome were combined together to enrich DEGs and DEMs on the metabolic pathways. The results aim to provide new insights into the molecular mechanism of feed conversion and assist with selective breeding to improve the feed efficiency of *M. nipponense*.

## Results

### RFI character analysis results

The average values of ADG and DFI of all test prawns were 0.0062 g a day, and 0.0282 g a day, respectively. The average RFI of all experimental animals was 0.0002 g/day. The estimated RFI values of the test prawns were -0.0075 to 0.0084 g/day (Supplemental Table S1).

The model used to estimate RFI was:

$$\mathrm{DFI}=0.039\mathrm{ MW}0.617+0.146*\mathrm{ADG}+\mathrm{e}$$

The ADG of individuals in HRFI was 0.0072 g/day, and the ADG value of LRFI group was the same as that of HRFI group. The coefficients of variation of ADG in HRFI and LRFI groups were 87.50% and 95.83%, respectively, indicating significant inter-individual variations in ADG. The mean RFI value of the HRFI group was 0.0050 g/day, and -0.0045 g/day in the LRFI group (Table 1).

**Table 1:** Statistics of average daily gain, daily feed intake, and residual feed intake of HRFI and LRFI groups from day 1 to day 75

Group	Parameter	Trait		
		**ADG**	**DFI**	**RFI**
HRFI	Mean	0.0072	0.0348	0.0050
	SD	0.0063	0.0087	0.0018
	CV	0.8750	0.2500	-
LRFI	Mean	0.0072	0.0250	-0.0045
	SD	0.0069	0.0078	0.0018
	CV	0.9583	0.3120	-

*ADG* Average daily gain (g/day), *DFI* Daily feed intake (g/day), *RFI* Residual feed intake (g/day), *Mean* Average value of trait estimation, *SD* Standard deviation of trait estimation, *CV* Estimated coefficient of variation of traits (%)

### Transcriptome analysis results

#### Sequencing and quality control of transcriptome data

A total of 77.81 Gb clean reads were produced after sequencing the hepatopancreas and muscles of *M. nipponense*. The Q30 base distribution was from 91.35 to 94.99%, and the average GC content was 46.90% (Table 2). The expression of *M. nipponense* protein-coding genes was analyzed according to the comparative results of HRFI and LRFI transcriptome sequencing.

**Table 2:** Statistics of the transcriptomic sequencing data of *M. nipponense* in the HRFI and LRFI groups

Group	Sample	No. Rawreads / M	Raw Bases /G	No. clean Reads / M	Clean Bases /G	Valid Bases /%	Q30 /%	GC /%
HRFI	Hepatopancreas_1	38.49	5.77	37.59	5.54	95.98	94.17	44.11
	Hepatopancreas_2	46.05	6.91	44.98	6.63	96.02	94.33	44.44
	Hepatopancreas_3	44.67	6.70	43.77	6.46	96.43	94.66	44.69
	Muscle_1	42.18	6.33	41.43	6.12	96.75	94.92	44.89
	Muscle_2	44.93	6.74	43.92	6.48	96.12	93.99	44.90
	Muscle_3	47.57	7.13	46.72	6.91	96.79	94.99	44.99
LRFI	Hepatopancreas_1	48.15	7.22	47.21	6.96	96.42	94.03	47.73
	Hepatopancreas_2	46.81	7.02	45.90	6.74	96.01	91.35	49.13
	Hepatopancreas_3	41.52	6.23	40.67	6.01	96.45	93.80	49.22
	Muscle_1	43.71	6.56	42.88	6.33	96.56	93.99	49.53
	Muscle_2	46.03	6.90	44.94	6.61	95.78	93.25	49.59
	Muscle_3	48.60	7.29	47.59	7.02	96.25	93.51	49.59

#### Differential gene identification

**Fig. 1:**
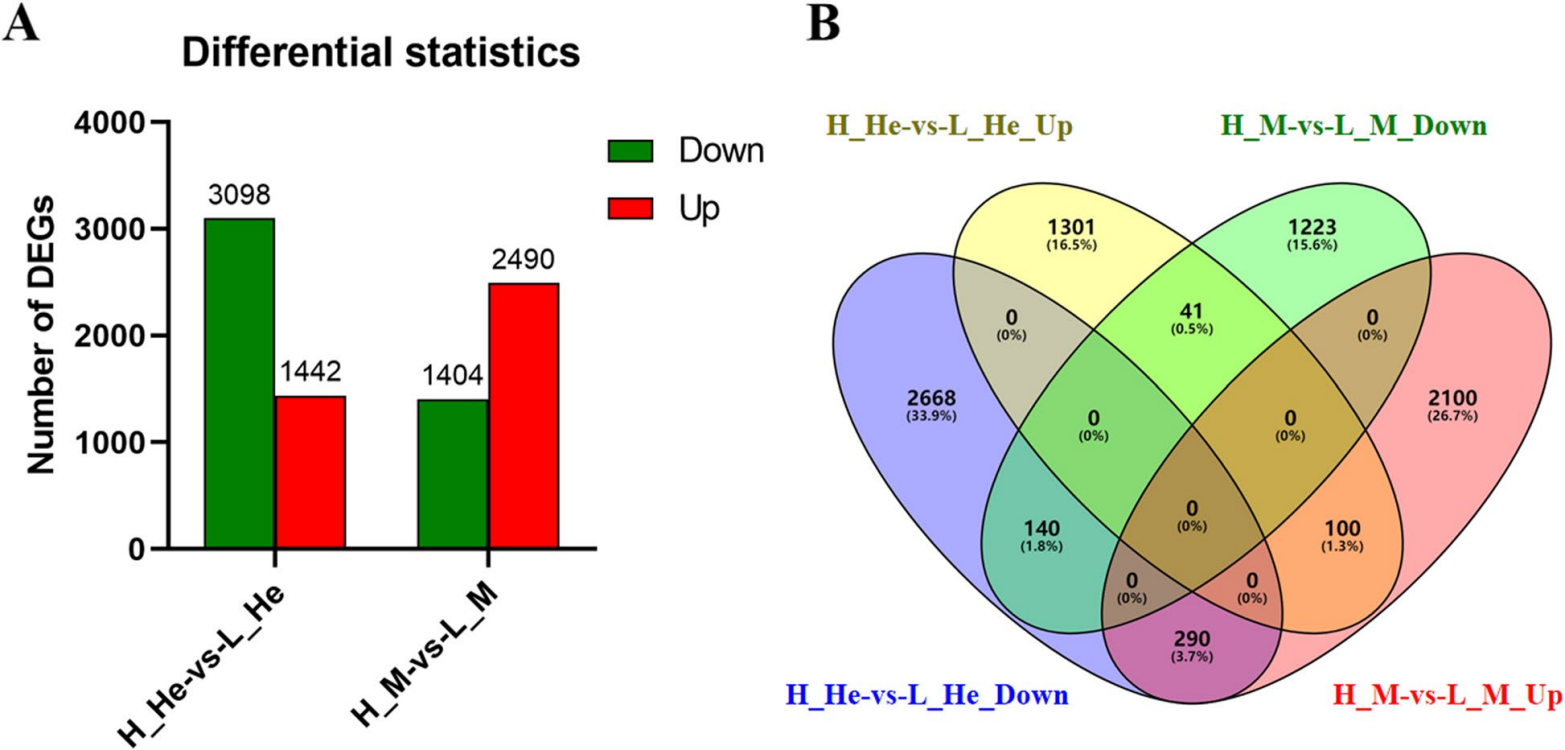
Differentially expressed genes (DEGs) analysis. Note: **A** DEGs in the H_He-vs-L_He and the H_M-vs-L_M. **B** Venn diagram of comparison of the DEGs

DEGs in the hepatopancreas and muscles of the HRFI and LRFI groups were identified, respectively. A total of 4540 (1442 up-regulated and 3098 down-regulated genes) transcripts in the hepatopancreas and 3894 (2490 up-regulated and 1404 down-regulated genes) transcripts in the muscle were found to be differentially expressed between the HRFI and LRFI groups (Fig. 1A). 571 identical genes were identified to be overlapped between the hepatopancreas and muscle transcriptome (Fig. 1B). Among the 571 DEGs, 141 genes were up-regulated and 430 genes were down-regulated in the hepatopancreas, and 390 gene were up-regulated and 181 were down-regulated in muscles between the HRFI vs LRFI group.

#### GO and KEGG enrichment analysis of differentially expressed genes

**Fig. 2:**
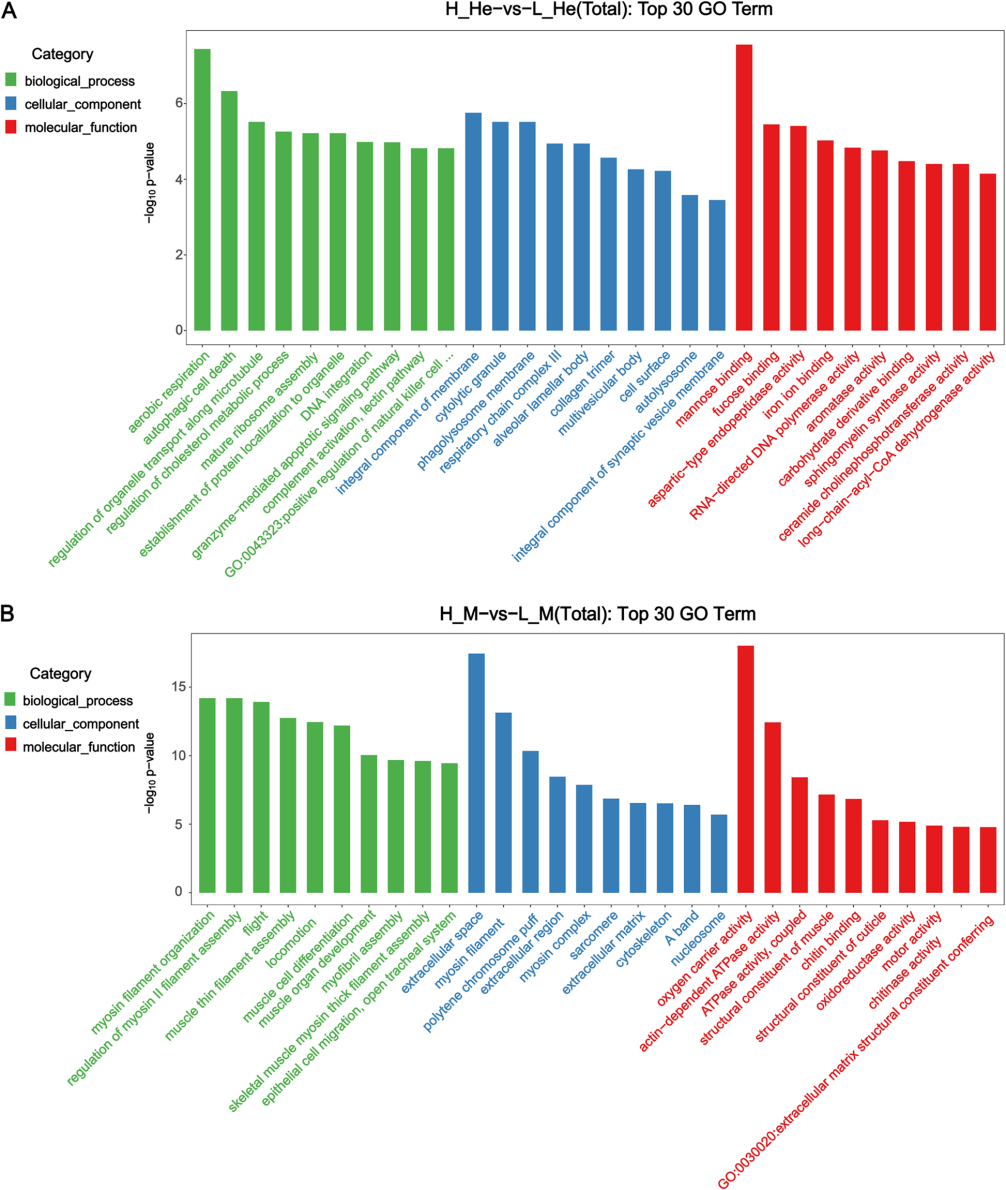
Gene ontology (GO) annotation analysis of DEGs in the hepatopancreas and muscle. Note: GO enrichment analysis top 30 (screen GO entries with the number of corresponding differential genes greater than 2 in the three classifications, and rank 10 entries in descending order according to the corresponding -log10 p-value of each entry) bar graph display. The horizontal axis in the figure is the name of GO entry, and the vertical axis is -log10 p-value. **A** H_He-vs-L_He (Total). **B** H_M-vs-L_M (Total)

To determine the functions of the DEGs related RFI, GO enrichment and KEGG pathway analyses were performed. The DEGs identified in this experiment covered all 3 domains: biological process, cellular component, and molecular function. GO enrichment showed that 366 GO terms were significantly enriched in hepatopancreas, including 220 terms in biological progress, 41 terms in cellular component, and 105 terms in molecular function. Also, 492 GO terms were significantly enriched in muscle, including 294 terms in biological progress, 95 terms in cellular component, and 103 terms in molecular function. Overall, GO terms with the most assigned genes were shown in Fig. 2. There were significant differences in GO terms in each domain for each tissue. It was discovered that there are substantial differences in GO terms of each domain in the hepatopancreas between the HRFI and LRFI groups. In the domain of biological process, the categories of catabolism process, synthetic process, cell cycle, and biological regulation were most abundant. For the domain of cellular component, the main subclasses were divided into cell nuclear part, organelle, plasma membrane and membrane part, and peripheral part of cell. In the domain of molecular function, most DEGs were related to enzymatic activity and binding. In the muscle, for the domain of biological process, regulation of muscle protein filament assembly, muscle development, cell cycle, and metabolic processes were the most abundant. In the domain of cellular component of GO, the main subclasses were divided into the extracellular region part, cytoplasm part, and cytoskeleton. In the domain of molecular function, most DEGs were related to the structural composition of muscle, binding, and the catalytic activity of enzymes.

**Fig. 3:**
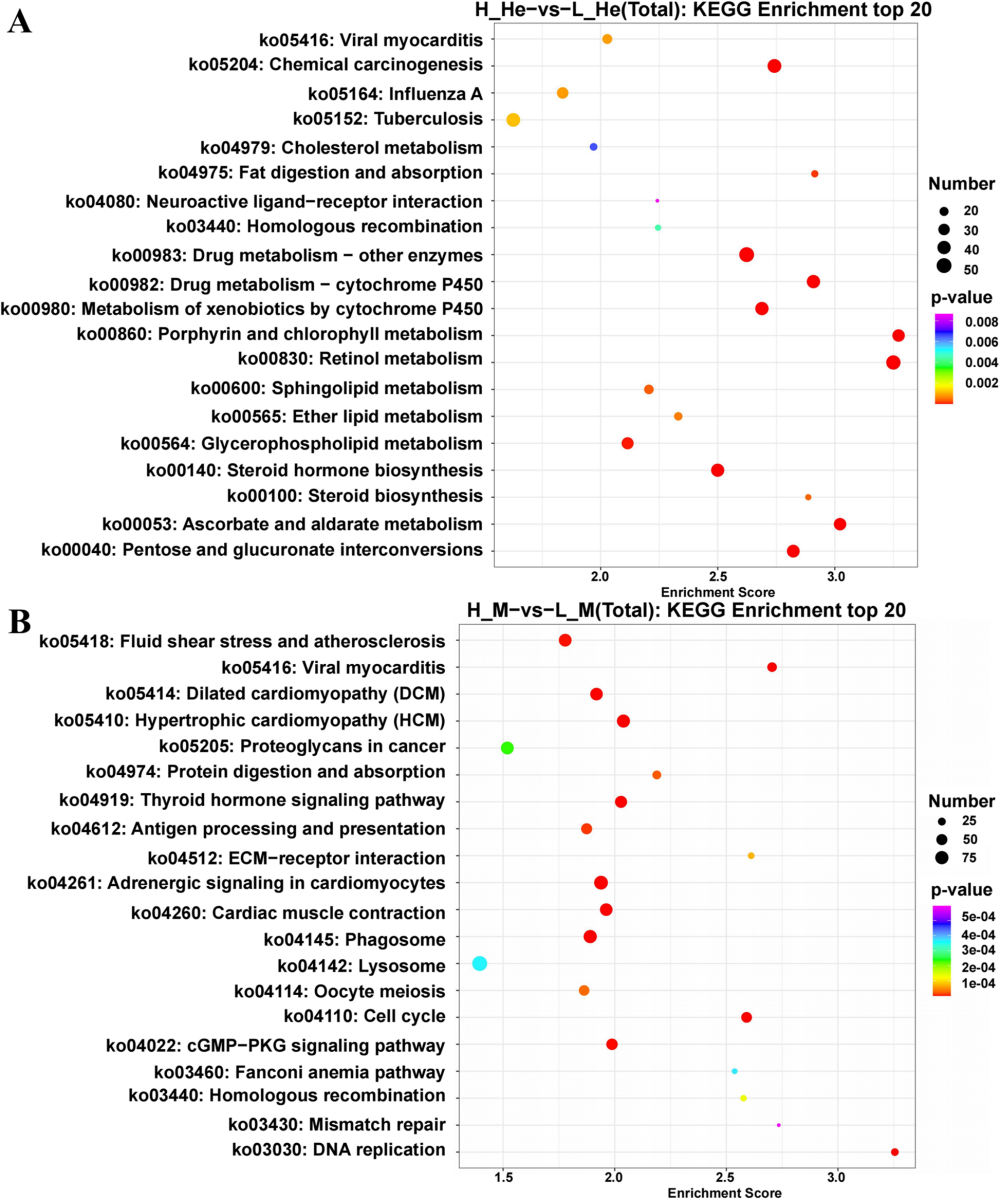
Kyoto Encyclopedia of Genes and Genomes (KEGG) analysis of DEGs in the hepatopancreas and muscle. Note: KEGG enrichment analysis top 20 (screening the Pathway entries with the number of corresponding differential genes greater than 2, and sorting by the corresponding -log10 p-value of each entry from large to small) bubble chart display. In the figure, the horizontal axis is the enrichment score. The items with larger bubbles contain more differential protein coding genes, and the bubble color changes from purple, blue, green to red. The smaller the enrichment p-value, the greater the significance. **A** H_He-vs-L_He (Total). **B** H_M-vs-L_M (Total)

The difference in the metabolic pathway between HRFI and LRFI groups was understood through the analysis of the KEGG pathway. These DEGs were mainly enriched into 47 KEGG pathways in the hepatopancreas and 106 KEGG pathways in the muscle. As shown in Fig. 3, the KEGG pathways enriched in the hepatopancreas between the HRFI and LRFI groups include viral myocarditis, chemical carcinogenesis, influenza A, tuberculosis, cholesterol metabolism, fat digestion and absorption, and homologous recombination, while DEGs in fluid shear stress and atherosclerosis, viral myocarditis, dilated cardiomyopathy, and hypertrophic cardiomyopathy were significantly enriched in the muscles between the HRFI and LRFI groups.

#### Differential gene expression between the HRFI group and LRFI group

Comparisons between the LRFI and HRFI groups of the hepatopancreas showed that the most significant DEGs include protein transport protein SEC13, retinol dehydrogenase 8, cytochrome P450 family 2 subfamily A6, alcohol dehydrogenase 1/7, glutathione S-transferase, diacylglycerol kinase (ATP) and alpha-amylase, etc. (Supplemental Table S2). These DEGs were involved in the metabolism of protein, exogenous substances metabolism, and energy production.

The DEGs significantly expressed in the muscle included isocitrate dehydrogenase, tubulin alpha, phosphoglucomutase, hexokinase, pyruvate kinase, glycogen synthase, and alpha-amylase (Supplemental Table S3). These DEGs were involved in energy production, muscle composition, and glucose metabolism. The commonly expressed DEGs between hepatopancreas and muscle were alcohol dehydrogenase 1/7, cytochrome P450 family 2 subfamily A6, Glucosamine-fructose-6-phosphate aminotransferase and protein transport protein Sec24C-like etc. The metabolism of exogenous substances, energy production, and protein metabolism pathways was significantly enriched by these DEGs (Supplemental Table S4).

#### qRT-PCR Validation of DEGs

**Fig. 4:**
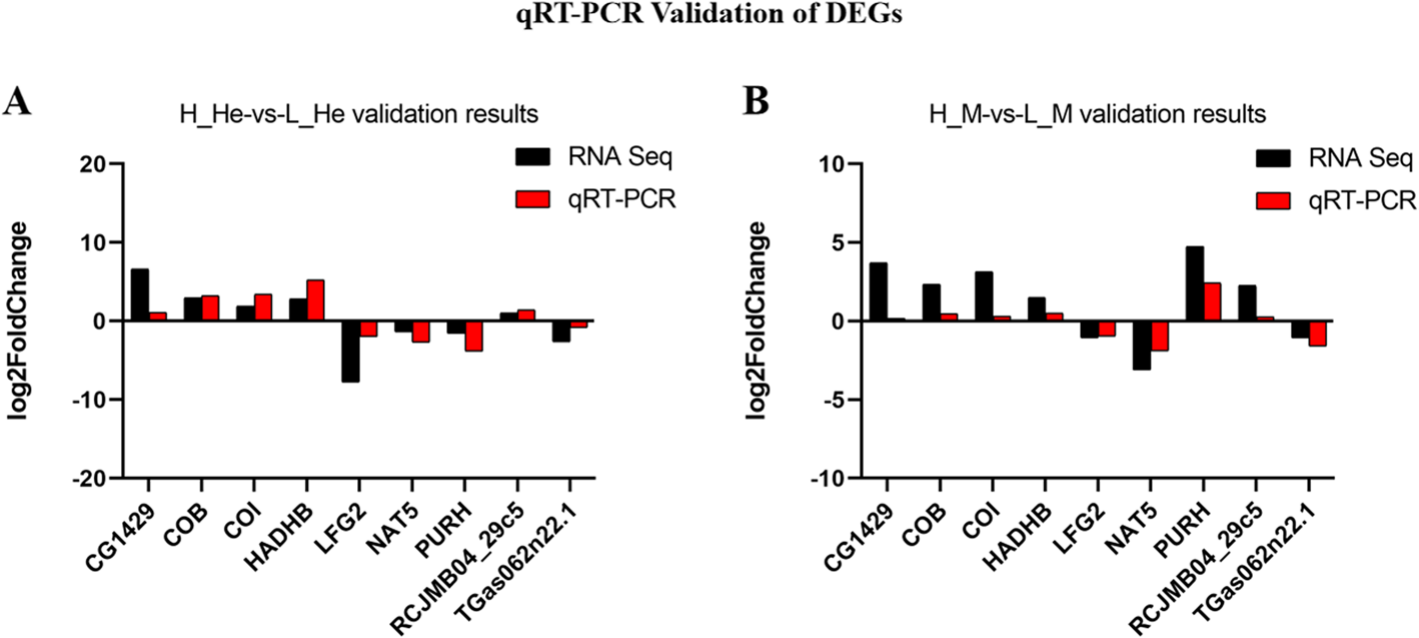
The expression of 9 DEGs in the transcriptome was verified by qRT-PCR. EIF was used as an internal reference gene to standardize the data, and fold changes were shown to demonstrate the RNA-seq results. Vertical bars represented the mean ± S.D (*n* = 3). Note: CG1429 (myocyte-specific enhancer factor 2), COB (cytochrome b), COI (cytochrome c oxidase subunit I, partial), HADHB (trifunctional enzyme subunit beta, mitochondrial-like), LFG2 (protein lifeguard 2-like isoform X2), NAT5 (N-alpha-acetyltransferase 20-like), PURH (putative bifunctional purine biosynthesis protein PURH), RCJMB04_29c5 (signal peptide peptidase-like 2A), TGas062n22.1 (ribosome maturation protein SBDS-like). **A** H_He-vs-L_He. **B** H_M-vs-L_M

Validation of RNA-Seq results by qRT-PCR. To verify the accuracy of RNA-seq detection, nine DEGs were randomly selected for qRT-PCR verification. Primers were designed based on mRNA sequences from RNA-seq data. Fold changes from qRT-PCR were compared with the RNA-Seq expression analysis results. The results showed that the expression patterns of the nine DEGs were consistent with the results of RNA-seq analysis, further demonstrating the reliability of transcriptome data (Fig. 4).

### Metabolome analysis results

#### Multivariate analysis

**Fig. 5:**
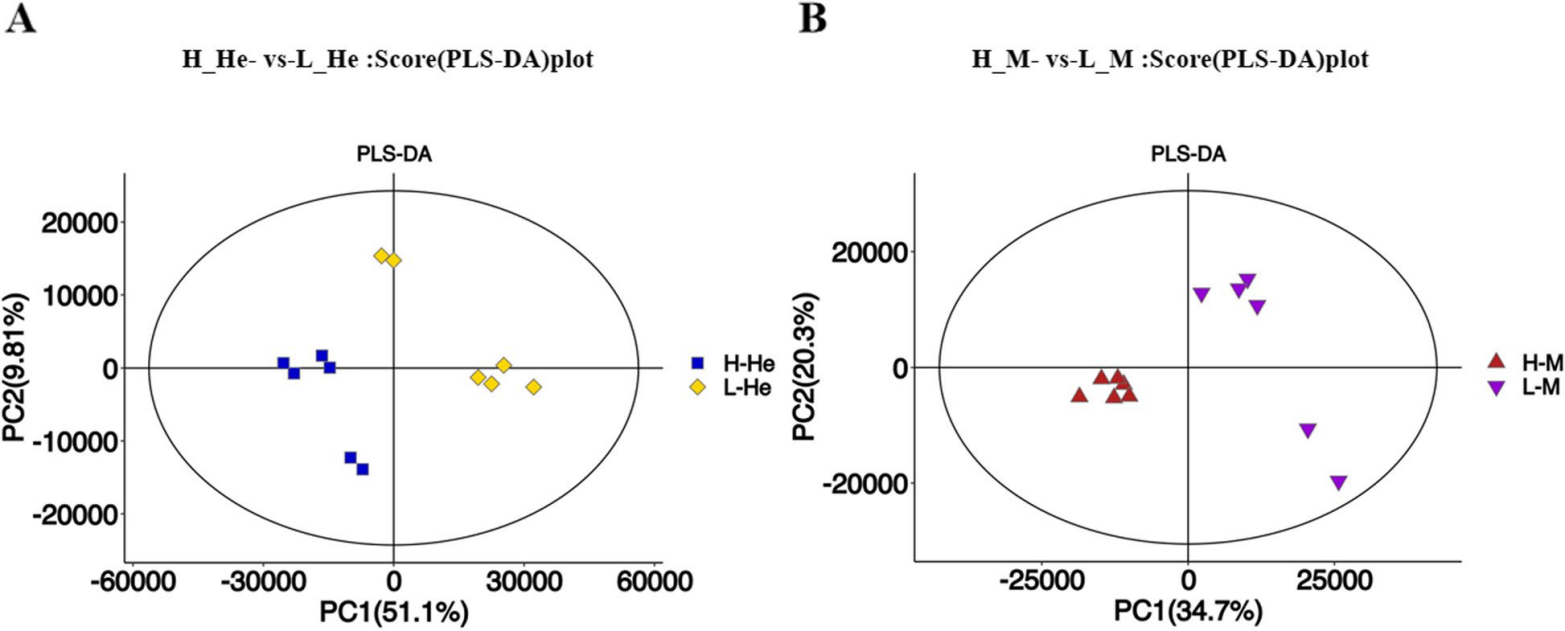
Partial least-squares discriminant analysis (PLS-DA) score plot. Note: **A** H_He-vs-L_He. **B** H_M-vs-L_M

**Fig. 6:**
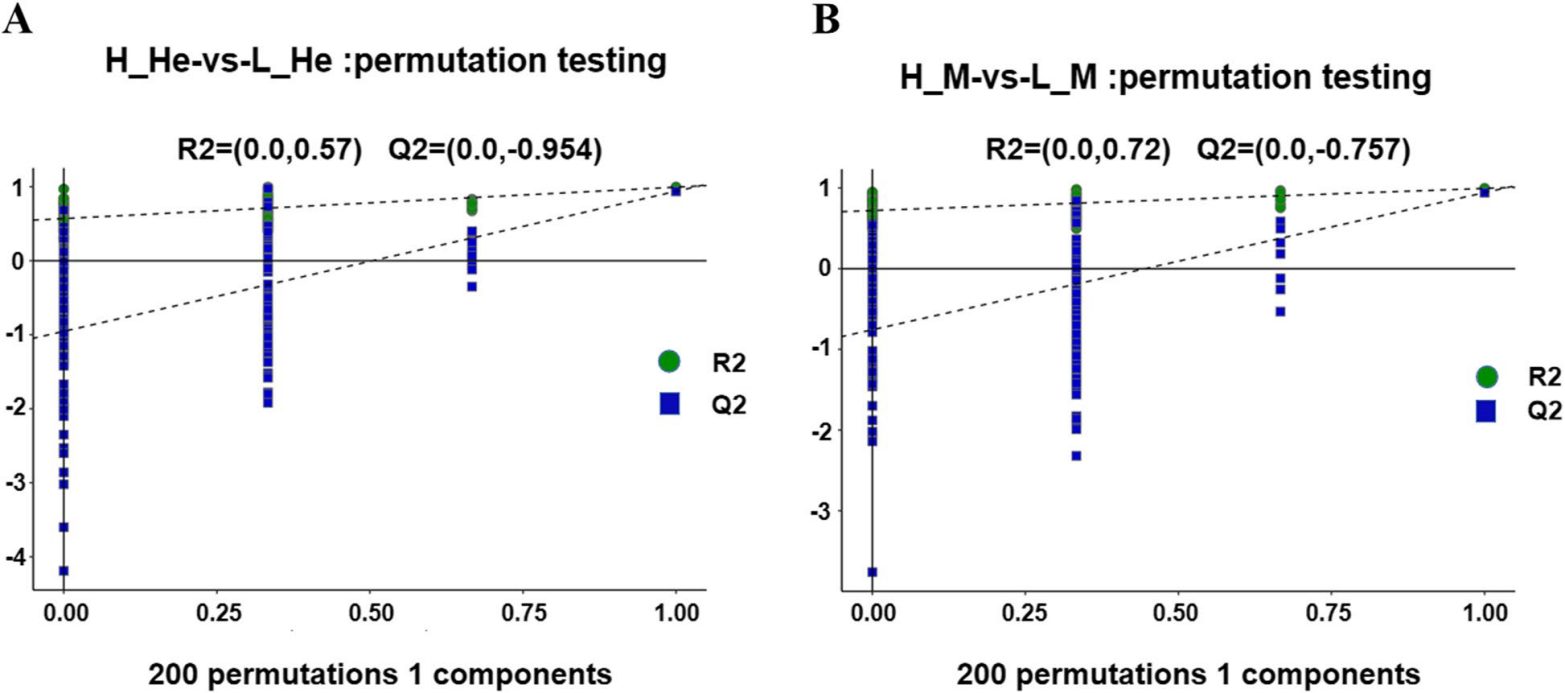
200 permutation tests performed on PLS-DA to obtain model verification of PLS-DA. (PLS-DA) score plot. Note: **A** The parameters of the R^2^ and Q^2^ scores were 0.57 and -0.954, respectively. H_He-vs-L_He. **B** The parameters of the R^2^ and Q^2^ scores were 0.72 and -0.757, respectively. H_M-vs-L_M

In order to maximize the difference in the hepatopancreas and muscle metabolome between the higher and lower RFI groups, the OPLS-DA model was used for multivariate statistical analysis to screen DEMs. The OPLS-DA analysis results showed that the HRFI and LRFI groups were significantly separated, indicating significant differences in metabolite profiles of hepatopancreas and muscle between the two groups. As shown in Fig. 5, the evaluation parameters of the hepatopancreas PLS-DA model were calculated as R^2^X = 0.68, R^2^Y = 0.994, and Q^2^ = 0.925. The evaluation parameters of the muscle PLS-DA model were calculated as R^2^X = 0.703, R^2^Y = 0.994, and Q^2^ = 0.965, indicating that the model was stable and reliable for subsequent analysis. As shown in Fig. 6, the slopes of the non-parallel lines of R^2^ and Q^2^ indicated that the OPLS-DA model was not over fitted. It showed that the model is stable and effective in fitness and prediction.

#### Principal component analysis of metabolite QC samples

In this study, LC–MS was used to analyze the DEMs of the hepatopancreas and muscles in *M. nipponense* among the HRFI and LRFI groups under long-term feeding conditions. A total of 6198 metabolites were identified in the hepatopancreas and muscle. In the hepatopancreas, 445 DEMs were identified (threshold VIP ≥ 1 and *p* < 0.05). Among the 445 DEMs, there were 164 amino acids and related derivatives (36.85%), 80 lipids and lipid-like molecules (17.98%), and 106 unclassified species (23.82%). Some major DEMs in the hepatopancreas were shown in Supplementary Table S5. In addition, 247 DEMs were identified in muscle (threshold VIP ≥ 1 and *p* < 0.05). Among the 247 DEMs, there were 37 amino acids and their derivatives (14.98%), 99 lipids and lipid-like (40.08%), and 48 unclassified species (19.43%). Some major DEMs in muscle were shown in Supplementary Table S6. There were 42 common DEMs detected in both the hepatopancreas and muscle. Among these DEMs, 15 were organic acids and derivatives, and 11 were lipids and lipid-like.

#### Enrichment analysis of KEGG metabolic pathway

**Fig. 7:**
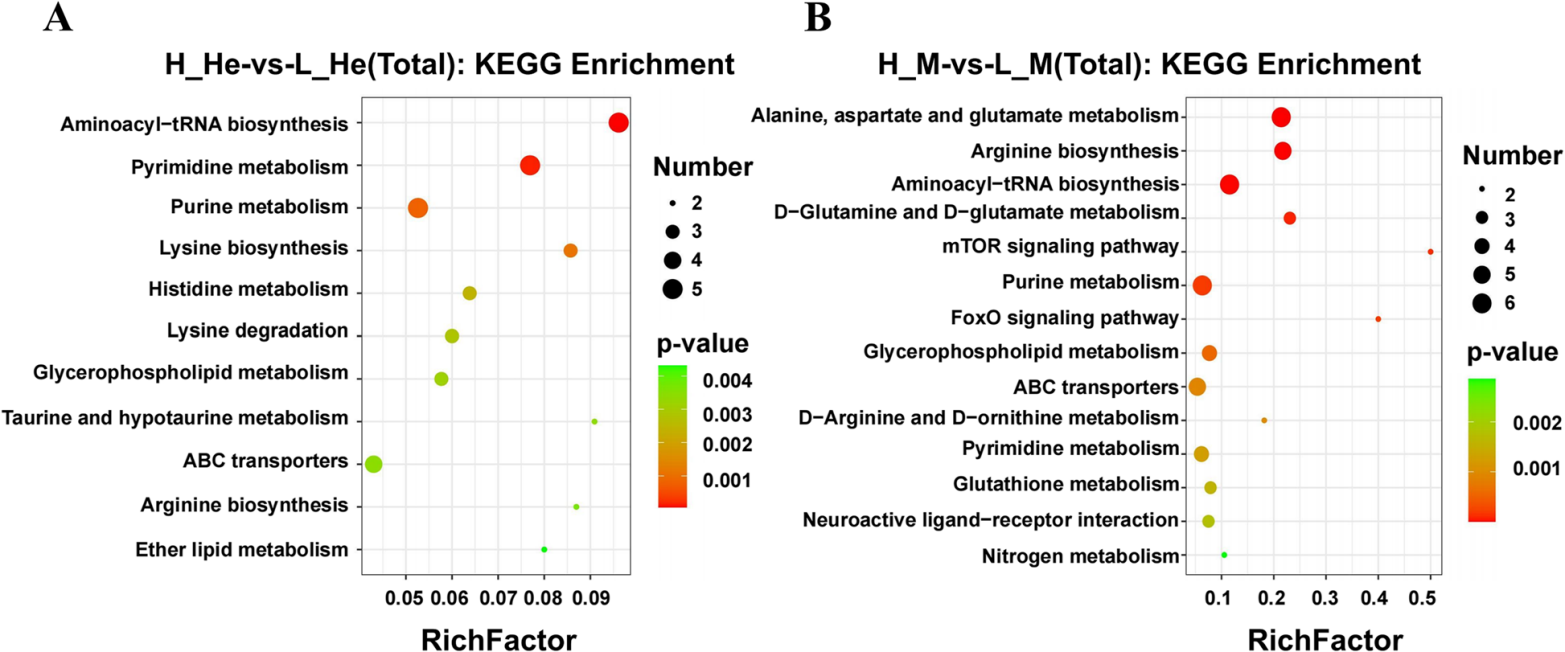
KEGG pathway enrichment analysis of the hepatopancreas and muscle of *M. Nipponense* after RFI extreme value grouping. Note: The abscissa of the bubble chart is the enrichment rate, and the ordinate is the KEGG pathway. The bubble size represents the number of metabolites enriched in the metabolic concentration in the path. The bubble color indicates the size of the P value, indicating enrichment significance. **A** H_He-vs-L_He. **B** H_M-vs-L_M

The enrichment analysis of differential metabolites was conducted based on the KEGG database. The most significantly enriched KEGG metabolic pathways were different between the tissues. As shown in Fig. 7, the most enriched KEGG metabolic pathways in the hepatopancreas were lysine biosynthesis, lysine degradation, purine metabolism, and pyrimidine metabolism. The most enriched KEGG metabolic pathways in the muscle were aspartate and glutamate metabolism, alanine, D − glutamine and D − glutamate metabolism, aminoacyl − tRNA biosynthesis, arginine biosynthesis, and mTOR signaling pathway.

#### Integrated analysis of transcriptome and metabolome

To determine the correlation between transcriptome and metabolome data, the KEGG pathways that were common in the two datasets were identified. 77 KEGG pathways were identified in the hepatopancreas and 93 in the muscle. Among them, 3 KEGG pathways, glycerophospholipid metabolism, ether lipid metabolism, and aminoacyl-tRNA biosynthesis, were enriched in both transcriptome and metabolome analysis of hepatopancreas. The glutathione metabolism pathway was enriched simultaneously in the two omics analyses of muscle. A total of 11 common DEMs and 41 related DEGs were recorded (Supplemental Table S7).

## Discussion

### Transcriptomic differences of hepatopancreatic and muscle between the HRFI and LRFI groups

Among the 7863 identified putative RFI-related DEGs, most DEGs with known functions participated in biological pathways related to immune response, digestive and absorption, nitrogen metabolism, carbon metabolism, lipid metabolism, etc. This result was similar to a study on *Litopenaeus vannamei*, such as cell cycle-yeast, glycolysis/gluconeogenesis, and ubiquitin-mediated proteolysis [18].

### Differences of xenobiotic metabolism and immune response between the HRFI and LRFI groups

Different responses of the body to external environment may lead to changes in RFI, which has been widely reported in terrestrial animals. For example, a report on Angus cattle in different RFI groups predicted that cattle with high feed efficiency were more sensitive to adverse environments [19]. Cytochrome P450 is mainly involved in the metabolism of endogenous and exogenous substances. [20]. In a previous study on transcriptomic analysis of bovine liver, cytochrome P450-related genes were found to be enriched [21]. In our research, Cytochrome P450-related genes were down-regulated in hepatopancreas (cytochrome P450 2L1-like isoform X1, X2, X3) and muscle (cytochrome P450 2L1-like, cytochrome P450 4C, cytochrome P450 9e2-like isoform X3 and cytochrome P450 V20) in the HRFI group. This may lead to a decrease in the capacity of *M. nipponense* to metabolize exogenous substances which ultimately leads to the variation in RFI. In crustaceans, normal aerobic metabolism will produce a certain amount of reactive oxygen species (ROS), which can play a vital role in the immune system [22]. However, excessive ROS can cause oxidative stress in the body, resulting in oxidative damage to cells [23]. Research showed that animals with low feed efficiency were more prone to oxidative stress [8]. Therefore, the body needs a robust antioxidant system to overcome the cell damage caused by oxidative stress. Our study showed that the expression of glutathione metabolism pathway-related genes, such as glutamate-cysteine ligase regulatory subunit-like (GCLC), 6-phosphogluconate dehydrogenase (G6PD), ribonucleoside-diphosphate reductase subunit M2 (RRM1), etc. was up-regulated in the muscle of the HRFI group, which could be to resist oxidative stress. It could be one of the reasons for the variation in RFI.

### Differences in lipid and protein metabolism between the HRFI and LRFI groups

It was found that body composition can affect the feed efficiency of animals. The energy requirements of protein and fat of the same weight are different, with protein consuming more energy [7]. A previous study showed that the down-regulation of trypsin expression would reduce protein utilization and deposition [24]. Similarly, in our study, the expression of hepatopancreatic trypsin-1 like protein and muscle carboxypeptidase A1 was down-regulated in the HRFI group, which may lead to the decrease of crude protein content in the hepatopancreas and muscle of *M. nipponense*. As reported, animals with high feed efficiency usually have high protein content [25], while animals with low feed efficiency tend to deposit more lipids [26]. In this study, several genes related to the fatty acid degradation pathway in the HRFI group were found to be down-regulated. Long-chain-acyl-CoA dehydrogenase (ACADL), a critical rate-limiting enzyme in the first step of the fatty acid oxidation [27], was down-regulated in the hepatopancreas. Others, including glutaryl-CoA dehydrogenase (GCDH), alcohol dehydrogenase class -3 (ALDH), and alcohol dehydrogenase 1/7 (ADH1_7), also showed a down-regulation trend in the hepatopancreas of HRFI group. Meanwhile, the fatty acid biosynthesis-related gene, estradiol 17-beta-dehydrogenase 8-like (H2-KE6), was up-regulated in the hepatopancreas of the HRFI group. Therefore, the differential expression of these genes would increase the crude lipid content in the hepatopancreas of *M. nipponense* in the HRFI group, possibly leading to the change of RFI.

### Differences in carbohydrate metabolism between the HRFI and LRFI groups

Carbohydrate metabolism in muscle can provide energy for prawn growth and physical activities. In our research results, KEGG pathways of carbohydrate digestion and absorption, such as glycolysis/gluconeogenesis, pentose phosphate pathway, starch and sucrose metabolism, etc., were significantly enriched. These pathways were relatively down-regulated in the muscles of the HRFI group. It is speculated that some changes in gene expression in these pathways may affect the efficiency of muscle energy utilization. Our study reported that the gene PFK9 encoding 6-phosphofructokinase, a critical enzyme in the glycolysis pathway, was significantly down-regulated in the glycolysis/gluconeogenesis pathway of muscle tissue in the HRFI group. ATP/AMP and H^+^ concentration have a significant effect on its activity. The gene was also involved in the fructose and mannose metabolism, pentose phosphate pathway, galactose metabolism, AMPK signaling pathway, and glucagon signaling pathway [28]. In addition, our study showed genes in the glycolysis/gluconeogenesis pathway, including glucose-6-phosphate isomerase (GPI), phosphoglycerate kinase (PGK), triosephosphate isomerase (TPI), lactate dehydrogenase (LDH), and pyruvate kinase (PK), were down-regulated. These genes were relatively down-regulated, resulting in a decrease in the carbohydrate utilization ability of *M. nipponense* in the HRFI group. The reduction of energy utilization efficiency could lead to RFI variation.

### Differences in other metabolism pathways between the HRFI and LRFI groups

Other common KEGG pathways enriched in the hepatopancreas and muscle were sphingolipid metabolism, homologous recombination, lysosome, other glycan degradation, and Mismatch repair. The differential expression of these KEGG pathways indicated that the factors affecting feed efficiency in different tissues and organs have certain commonalities. It also suggested that no single mechanism can dominate the changes in RFI phenotype [7].

### Metabolomic differences of hepatopancreatic and muscle between the HRFI and LRFI groups

In this study, LC–MS was used to analyze the DEMs in the hepatopancreas and muscle of *M. nipponense* between extreme RFI groups. Many metabolic changes in the hepatopancreas and muscle of *M. nipponense* lead to differences in feed efficiency, including lipid metabolism, amino acid metabolism, nucleotide metabolism etc. Among the DEMs screened in this study, there were more lipid substances enriched in *M. nipponense* in the HRFI group, while there were more amino acids and amino acid derivatives enriched in *M. nipponense* in the LRFI group. In rainbow trout (*Oncorhynchus mykiss*), the difference in protein and lipid contents between different RFI groups have also been reported which were related to the additional energy costs of fat and protein deposition [12].

### Differences of immune metabolites between the HRFI and LRFI groups

Research has shown that animals with low RFI value (high feed efficiency) have weak immunity as they save energy with an inadequate immune response in order to improve feed efficiency [29]. Aspirin or acetylsalicylic acid (acetosal) is a drug in the salicylate family. It is usually used as an analgesic, antipyretic and anti-inflammatory drug. It also has anticoagulant effects [30]. The up-regulated expression of Aspirin was detected in both hepatopancreas and muscles of the HRFI group. This result suggested that the immune response of *M. nipponense* in the HRFI group was more robust. Studies have shown that excessive or long-term immune response would reduce the synthesis of skeletal muscle and systemic growth of pigs and decrease feed efficiency [31]. In our study, the differential expression of the glutathione metabolic pathway in the muscle of the HRFI group was detected in the transcriptome and metabolome analyses, which further confirmed that animals with low feed efficiency would spend more energy on the immune response, leading to changes in RFI.

### Differences of amino acid and lipid metabolites between the HRFI and LRFI groups

The KEGG pathway enrichment analysis results showed significant differences in lysine biosynthesis and lysine degradation pathways between the hepatopancreas of the HRFI and LRFI groups. In organisms that synthesize lysine, the metabolic pathway of lysine is affected by the metabolite 2-Aminoadipate [32]. Lysine is a restrictive amino acid that plays various roles in organisms. Lysine impacts growth and immunity [33]. In this study, the expression of lysine in the hepatopancreas and muscle of the HRFI group was up-regulated. Lysine also cross-links collagen peptides, absorbing mineral elements, and producing carnitine, which is the key to fatty acid metabolism [34]. Short-chain acylcarnitine is an intermediate of glucose, amino acid, and fatty acid metabolism, while long-chain and medium-chain acylcarnitine only come from the fatty acid metabolism [35]. The acylcarnitine transports the acyl functional group of organic acids and fatty acids from the cytoplasm to the mitochondria, so they can be broken down to generate energy. This reaction step is called β- Oxidation. Acylcarnitine can accumulate in tissues. The distribution of acylcarnitine can be used as an indicator of energy metabolism. At the same time, acylcarnitine can also be used as a molecular marker for incomplete fatty acid oxidation. The low accumulation of these intermediates in cells may indicate that the complete oxidation through the tricarboxylic acid (TCA) cycle is more efficient, hence more energy is extracted from oxidation [36]. In this study, six acylcarnitines were highly expressed in the muscles of the HRFI group, indicating that the energy utilization efficiency of *M. nipponense* in the muscle of the LRFI group was higher. This result further indicated that animals with lower RFI had higher feed efficiency. Although five acylcarnitines were also found in the hepatopancreas, three were down-regulated, and two were up-regulated in the HRFI group. The differential expression between different tissues may be due to the apparent preference of metabolic tissues for substrate oxidation and the difference in the utilization efficiency [37], which is worth further study.

### Transcriptomic and Metabolomic differences integration analysis of hepatopancreatic and muscle between the HRFI and LRFI groups

The feed conversion efficiency of *M. nipponense* was correlated with different genes and metabolites in the hepatopancreas and muscle. This study found that some of the same pathways were enriched in the two omics datasets. Notably, glycerophospholipid metabolism, ether lipid metabolism, and aminoacyl-tRNA pathways were enriched in the comprehensive hepatopancreas tissue analysis (Supplemental Table S7).

### Differences in protein and lipid KEGG pathways enriched between the HRFI and LRFI groups

The physiological function of Aminoacyl-tRNA synthetases (ARSs) is to catalyze the connection between transfer RNAs and their corresponding amino acids. They are essential enzymes for protein synthesis in all biological cells [38]. Aminoacyl-tRNA participates in the processing and translation of genetic information. Its biosynthesis is characterized by the chemical bonding between tRNA and its amino acids during protein synthesis. The rate at which genetic information is translated will affect the feed efficiency of fish [39]. In a metabolomic analysis of the feed efficiency of cattle, it was found that the aminoacyl-tRNA biosynthesis pathway was also enriched [40]. In our study, the up-regulation of L-lysine, L-proline, and L-isoleucine and their corresponding genes lysyl-tRNA synthetase, class I, prolyl-tRNA synthetase, and isoleucyl-tRNA synthetase in HRFI group may promote the production of proteins (Figure S1). Therefore, the differential expression of the aminoacyl-tRNA biosynthesis pathway may have a specific impact on feed efficiency.

Both glycerophospholipid metabolism and ether lipid metabolism pathways belong to lipid metabolism, and the down-regulated of related genes in these two metabolic pathways further explains the down-regulated of the ability of the hepatopancreas to degrade lipids in the HRFI group. And resulted in an up-regulated of glycerophosphate choline and glycerophosphate ethanolamine. This can lead to variations in RFI.

### Differences of Glutathione metabolism pathway between the HRFI and LRFI groups

Notably, glutathione metabolism was found to be enriched in a comprehensive analysis of muscle. Gamma-glutamyltranspeptidase (GGT), also known as glutathione hydrolase, catalyzes the transfer of the glutamyl functional group of glutathione to an acceptor, which might be a dipeptide receptor or a variety of amino acids [41]. GGT releases free glutamate and dipeptide cysteinyl-glycine from the metabolism of glutathione. Glutamate is a non-essential amino acid that can participate in energy generation through gluconeogenesis [42]. Some studies have shown that glutamate can improve the feed efficiency of pigs [43]. In this study, the GGT gene and L-glutamate were up-regulated (Figure S2). Hence, the differential expression of the glutathione metabolism pathway might specifically impact feed efficiency.

## Conclusion

In this study, the analyses of transcriptomes and metabolomes in the hepatopancreas and muscle of *M. nipponense* from high and low RFI groups conducted here were combined to explore the mechanism of nutrient feed conversion efficiency. In the transcriptome analysis, the differences in immune response (cytochrome P450 related genes, glutathione metabolism pathway), lipid and protein metabolism (trypsin-1-like, fat acid degradation pathway), and energy metabolism (PFK9, glycolysis/gluconeogenesis pathway) related genes and metabolic pathways could affect the feed conversion efficiency of *M. nipponense*. Metabolomic analysis showed that the differential expression of amino acids, lipids, and immune substances represented by lysine, acylcarnitine, and aspirin could affect the feed conversion efficiency of *M. nipponense*. In addition, multi-omic analysis of transcriptomics and metabonomics indicated that differences in lipid metabolism, amino acid metabolism (aminoacyl-tRNA biosynthesis pathway), and immune response (glutathione metabolism pathway) might lead to changes in RFI. These DEGs and DEMs provide a reference for understanding the various mechanisms of feed efficiency levels at the molecular level.

## Materials and methods

### Experimental materials and method

The larvae of *M. nipponense* were obtained in Wuyi County, Zhejiang Province, China. The experiment was performed at the Hangzhou Fisheries Research Institute (Hangzhou, China) from July to October 2021.

The larvae of *M. nipponense* were cultured for a one-week in the experimental environment before selection. All prawns were raised under the same culture conditions and feeding scheme. Ultimately, 72 M*. nipponense* (Average body weight of 0.3367 ± 0.0816 g) were used in the feeding experiment. The experiment (water volume: 37L, temperature: 27 ± 1 °C) was carried out in 18 glass tanks (1000 mm × 500 mm × 350 mm) respectively. 4 prawns were raised in each glass tank and separated into individual segments by a clear plastic plate. In the feeding experiment, the prawns were fed with the commercially formula pellet diet (Tongwei Feed Group CO, Ltd.), which remain stable as pellets for at least one day, twice daily (9:00 a.m. and 7:00 p.m) for 75 days, and an appropriate feed dose per meal was ensured for their apparent satiation. The total feed for each prawn was stored in a separate sealed bag. The food that was not eaten was collected daily in a separate container at 7:30 a.m and transferred into a -80℃ refrigerator. At the end of the experiment, the uneaten feed was uniformly dried until the weight was stable. During the experiment, 10% of the water in the tank was changed every two days, and dead prawns and molts were removed promptly.

### Data collection and RFI calculation

The total feed consumption of each prawn was calculated. The unconsumed feed for each prawn was collected daily during the experimental period and recorded. Feed intake (FI) was calculated as the difference between the total amount of feed consumed and not consumed. The initial weight (BW1) (g) and final weight (BW2) (g) of each prawn at the beginning and end of the experiment were recorded, and the weight gain (WG) (g) during the whole experiment was calculated. The ADG (g / d) and daily feed intake (DFI) (g / d) were calculated as WG and FI divided by the number of days of culture (75 days) during the entire experimental period, respectively.

A total of 55 prawns were used for the RFI experiment. To calculate RFI, DFI (calculated from phenotypic traits) was taken as the dependent variable, and ADG was taken as the independent variable for nonlinear multiple regression calculation [2]:

$$\mathrm{DFI }=\mathrm{ b}1*\mathrm{MWb}2 +\mathrm{ b}3*\mathrm{ADG }+\mathrm{ e}$$

where MW is the mid-weight (MW = 1 / 2 (BW1 + BW2)), MW^b2^ is the medium metabolism amount. As mentioned above, DFI is the daily feed intake, ADG is the average daily gain, b1, b2, and b3 are regression coefficients of related variables, e is the error term, which is considered as RFI.

The calculation of various correlation coefficients (b1, b2, and b3) in this equation is completed by the nonlinear regression program of NLS in R 4.1.1 software.

### Transcriptome analysis

According to the calculated RFI, 15 prawns with the highest RFI value were selected as the highest RFI group (HRFI), and 15 prawns with the lowest RFI value were chosen as the lowest RFI group (LRFI). Every two individuals of 15 prawns with the RFI mean value close to intra-group mean value were selected to construct three parallel samples in the HRFI and LRFI groups, respectively. Six prawns respectively from the HRFI and LRFI groups were selected for comparative transcriptomic analysis.

Prawns were dissected after anesthesia on ice, and tissue samples from the muscles and hepatopancreas were collected and stored in − 80 °C for further downstream analyses. A total of 24 samples (i.e., 6 high RFI and 6 low RFI groups × 2 tissues per prawn) were collected. For each tissue, an equal amount of samples from the 6 prawns in each group were pooled into 3 tubes for total RNA extraction. Hence, there were a total of 12 pooled samples (i.e., 3 high RFI + 3 low RFI × 2 tissues). Total RNA was extracted using Trizol following standard protocol. The concentration of the RNA was then determined with NanoDrop 2000 spectrophotometer (Thermal Science, USA). The integrity of RNA was evaluated using Agilent 2100 Bioanalyzer (Agilent Technologies, CA, USA). The libraries were constructed with TruSeq chain mRNA LT Sample Prep Kit (Illumina, CA, USA) according to manufacturer’s instructions.

Sequencing of the libraries were performed on IlluminaHiSeqXT0n platform, to generate 150 bp of terminal sequences. Raw reads were converted to fastq format using Trimmomatic [44], and low-quality reads were removed. The processed clean reads were used for subsequent analysis. The clean reads were mapped to the genome of *M. nipponense* (cngb_100843) [45] using HISAT2 [46]. Cufflinks [47] was used to calculate the FPKM of each gene [48], and the read counts of each gene were obtained with HTSeqcount [49]. Differential expression between genes was analyzed using DESeq (2012) in the R software package [50]. Fold change (FC) > 2 or FC < 0.5 and *P* value < 0.05 were taken as the threshold of significant differential expression. Hierarchical cluster analysis was performed on DEGs to demonstrate gene expression patterns in different groups and samples. Based on the hypergeometric distribution, the software R was used to carry out the GO enrichment and KEGG pathway [51] enrichment analysis of DEGs.

Reads were assembled using StringTie [52], and the software Cuffcompare was used to compare reference genomes and known annotation genes, gene structure expansion, and identify new transcripts.

### Metabolome analysis

Metabolites were extracted from the hepatopancreas (*n* = 6) and muscle (*n* = 6) samples from each RFI group (HRFI/LRFI). In brief, 30 mg of the test sample was frozen and pulverized into a fine powder without defrosting (60 Hz, 2 min). The hepatopancreas or muscle samples were extracted using 267 µL methanol and water (4 / 1, vol/vol) in an ultrasonic ice bath for 10 min. The samples were frozen at—20 ℃ for 30 min. After a final centrifugation step (4 ℃, 13,000 rpm, 10 min), the solvent extract (150 µL) was transferred to LC vials. The vials were stored at—80 ℃ until ready for LC–MS analysis. L-2-Chlorophenylalanine (13 μL, 0.06 mg / mL) was dissolved in a methanol solution and used as the internal standard of this experiment. QC samples are prepared by mixing aliquots of all samples.

For chromatographic separation, a Dionex Ultimate 3000 RS UHPLC equipped with a Q-Exactive was used coupled to a quadrupole-Orbitrap mass spectrometer equipped with a heated electric spray ionization source (Thermo Fisher Scientific, Waltham, MA, USA) [53].

The Progenisis QI V2.3 (Nonlinear, Dynamics, Newcastle, UK) software was used to standardize the original data. The main parameters were 5 ppm precursor tolerance, 10 ppm product tolerance, and 5% production threshold. Qualitative analysis of detected compounds was performed using the Human Metabolome Database (HMDB), Lipid maps (V2.3), Metlin, EMDB, PMDB, and self built database. Principal component analysis (PCA) was performed using R software to observe the overall distribution and stability of the sample. Partial Least-Squares-Discriminant Analysis (PLS-DA) and Orthogonal Partial Least-Squares-Discriminant Analysis (OPLS-DA) were used to distinguish metabolites from different groups. To prevent over fitting, sevenfold cross-validation and 200 Response Permutation Testing (RPT) were used to evaluate the model quality of the experiment.

### Validation of expression levels using qRT-PCR

CG1429 (myocyte-specific enhancer factor 2), COB (cytochrome b), COI (cytochrome c oxidase subunit I, partial), HADHB (trifunctional enzyme subunit beta, mitochondrial-like), LFG2 (protein lifeguard 2-like isoform X2), NAT5 (N-alpha-acetyltransferase 20-like), PURH (putative bifunctional purine biosynthesis protein PURH), RCJMB04_29c5 (signal peptide peptidase-like 2A), TGas062n22.1 (ribosome maturation protein SBDS-like) were selected for qRT-PCR analysis to verify the reliability and accuracy of transcriptomic data. A total of 9 primer pairs were designed for both hepatopancreas and muscle transcripts that were differentially expressed. The primer sequence and product size are shown in Table 3. Reverse transcription was carried out using the PrimeScript RT reagent Kit (TaKaRa) according to the manufacturer’s instructions. Quantitative Real-Time PCR (qRT-PCR) was carried out on a CFX96 Real-time PCR Detection System (BioRad, Hercules, CA, USA), and SYBR® Green was used as fluorescent dye. The qRT-PCR reactions were carried out in triplicates for each tissue of two groups, each 20 μL reaction volume containing 10 μL SYBR Green Master Mix (TaKaRa, Shanghai, China), 0.8 μL of each primer of concentration 10 μM, 1.6 μL cDNA of concentration 200 ng/μL and 6.8 μL of RNase-free water. The qRT-PCR conditions were as follows: 95 °C for 10 s followed by 40 cycles of 95 °C for 15 s, 55 °C for 30 s, and 72 °C for 30 s. Quantification of relative gene expression was calculated using the 2^−ΔΔct^ method using the reference gene (EIF) to normalize the relative expression. [54].

**Table 3:** Primers used to verify transcriptome data

Gene name	Primers (5’-3’)
CG1429	F: GCCTCCTGTGTTTGTGAATC
	R: CTCCTATCTCCGCAAAGTGT
COB	F: TCCTAACAAACTAGGCGGAG
	R: TGTATGGGTCTTCTACGGGT
COI	F: AGTAGCACACTTCCACTACG
	R: GTTGAGTGATAGGCCGGTAA
HADHB	F: GCTAAAGAAATGGGCTGGAA
	R: TTGGCAAGAACCTGACCTG
LFG2	F: CACTGGCTGTGGAATCTACT
	R: CCATCTGCGTATCGTACACT
NAT5	F: ATATCGTCGACTTGGTCTGG
	R: TGAAGAACAACGCGATACAC
PURH	F: GATCTTGGGGAACTTACTCC
	R: CACCATCAGATACCTCCCTA
RCJMB04_29c5	F: GGATACTTAACACTCGGCCA
	R: GAGGGCATCGGATATTTGGA
TGas062n22.1	F: CTACCGAACAATCGAAGA
	R: GCAGTATCAAATGGCAAC
EIF	F: CATGGATGTACCTGTGGTGAAAC
	R: CTGTCAGCAGAAGGTCCTCATTA

The experimental data were recorded using the mean ± standard deviation method of the three repeats. All data were analyzed by a one-way ANOVA. The relative expression level of the 9 DEGs were compared and regarded as statistically significant when *p* value is < 0.05.

## Supplementary Information

**Additional file 1:** **Table S1.** Descriptive statistics ofaverage daily gain, daily feed intake and residual feed intake for 1-75 days.

**Additional file 2:** **Table S2.** Description of the DEGs of the HRFI and LRFIgroups in hepatopancreas.

**Additional file 3:** **Table S3.** Description of the DEGs of HRFI and LRFI groupsin muscles.

**Additional file 4:** **Table S4.** Description of DEGs shared by the twoexperimental groups.

**Additional file 5:** **Table S5.** Description of DEMs of the HRFI and LRFI groupsin hepatopancreas.

**Additional file 6:** **Table S6.** Description of DEMs of HRFI and LRFI groups inmuscles.

**Additional file 7:** **Table S**7. Comprehensive analysis of the metabolic enrichment pathways bymulti-omics.

**Additional file 8:** **FigureS1.**

**Additional file 9:** **FigureS2.**

## Data Availability

All data and materials supporting our findings are included in the manuscript. The datasets supporting the conclusions of this article are available as follows: Sequence reads from the RNA-Seq experiment are available from the NCBI https://www.ncbi.nlm.nih.gov/sra/PRJNA908147 Metabolomics data have been deposited to the EMBL-EBI MetaboLights database (https://doi.org/10.1093/nar/gkz1019, PMID:31,691,833) with the identifier MTBLS6663. The complete dataset can be accessed here.https://www.ebi.ac.uk/metabolights/MTBLS6663
