# Supplementary material for: Combined transcriptome and metabolome analysis reveal key regulatory genes and pathways of feed conversion efficiency of oriental river prawn Macrobrachium nipponense
Source: BMC Genomics. 2023 May 19;24:267. doi: 10.1186/s12864-023-09317-1 (PMC10197838; doi:10.1186/s12864-023-09317-1)
Supplement: Supplementary file 1 — Additional file 1: Table S1. Descriptive statistics ofaverage daily gain, daily feed intake and residual feed intake for 1-75 days. [file 12864_2023_9317_MOESM1_ESM.docx]

**Table S1 Descriptive statistics of average daily gain, daily feed intake and residual feed intake for 1-75 days.**

| **Parameter** | **Trait** | | |
| --- | --- | --- | --- |
|  | **ADG** | **DFI** | **RFI** |
| Max | 0.0280 | 0.0626 | 0.0084 |
| Min | 0.0007 | 0.0155 | -0.0075 |
| Mean | 0.0062 | 0.0282 | 0.0002 |
| SD | 0.0059 | 0.0086 | 0.0038 |
| CV | 0.9516 | 0.1374 | - |

Note: Max: trait estimation, Min: minimum value of trait estimation, Mean: average value of trait estimation, SD: standard deviation of trait estimation, CV: estimated coefficient of variation of traits (%).
