# Supplementary material for: Combined transcriptome and metabolome analysis reveal key regulatory genes and pathways of feed conversion efficiency of oriental river prawn Macrobrachium nipponense
Source: BMC Genomics. 2023 May 19;24:267. doi: 10.1186/s12864-023-09317-1 (PMC10197838; doi:10.1186/s12864-023-09317-1)
Supplement: Supplementary file 2 — Additional file 2: Table S2. Description of the DEGs of the HRFI and LRFIgroups in hepatopancreas. [file 12864_2023_9317_MOESM2_ESM.docx]

| **Table S2 Description of the DEGs of the HRFI and LRFI groups in hepatopancreas.** | | | | |
| --- | --- | --- | --- | --- |
| **Gene Symbol** | **Description** | **Regulation** | **Gene Id** | **KEGG Pathway** |
| RDH8 | retinol dehydrogenase 8 | Down | MSTRG.1418.1; MSTRG.1418.2; MSTRG.15126.6;  MSTRG.15128.5; MSTRG.19284.5; MSTRG.4220.2;  MSTRG.5351.1 | ko00830 |
| CYP2A6 | cytochrome P450 family 2 subfamily A6 | Down | MSTRG.1242.1; MSTRG.1243.1 | ko00830, ko04726, ko05204 |
| ADH1_7 | alcohol dehydrogenase 1/7 | Down | MSTRG.14541.1; MSTRG.14541.4; MSTRG.14542.3 | ko00010, ko00071, ko00350, ko00830, ko00980, ko00982, ko05204 |
| GST | glutathione S-transferase | Down | MSTRG.17332.1; MSTRG.2911.3; MSTRG.2911.4; MSTRG.2912.2 | ko00480, ko00980, ko00982, ko00983, ko01524, ko04212, ko05200, ko05204, ko05225, ko05418 |
| ALDH | aldehyde dehydrogenase (NAD+) | Down | MSTRG.17879.1 | ko00010, ko00053, ko00071, ko00260, ko00280, ko00310, ko00330, ko00340, ko00380, ko00410, ko00561, ko00620 |
| AKR1A1 | alcohol dehydrogenase (NADP+) | Down | MSTRG.19900.1 | ko00010, ko00040, ko00561 |
| DGKA, DGK | diacylglycerol kinase (ATP) | Down | MSTRG.24291.10 | ko00561, ko00564, ko04070, ko04072, ko04361, ko05231 |
| MYLIP, MIR | E3 ubiquitin-protein ligase MYLIP | Down | MSTRG.14026.13 | ko04979 |
| LIPF | gastric triacylglycerol lipase | Down | MSTRG.14025.2; MSTRG.2424.1; MSTRG.25421.1; MSTRG.25421.3; MSTRG.8857.1; MSTRG.17562.1 | ko00100, ko00561, ko00564, ko00565, ko00590, ko00591, ko00592, ko04014, ko04270, ko04972, ko04975, ko04977 |
| PLA2G, SPLA2 | secretory phospholipase A2 | Down | MSTRG.2424.1; MSTRG.25421.1; MSTRG.25421.3; MSTRG.8857.1 | ko00564, ko00565, ko00590, ko00591, ko00592, ko04014, ko04270, ko04972, ko04975 |
| AMY, AMYA, MALS | alpha-amylase | Down | MSTRG.22498.1 | ko00500, ko04972, ko04973 |
| LIP, TGL2 | triacylglycerol lipase | Down | MSTRG.21663.10; MSTRG.21663.11; MSTRG.21663.4; MSTRG.14025.2; MSTRG.17562.1 | ko00100, ko00561, ko04714, ko04923, ko04972, ko04975, ko04977 |
| SGMS | sphingomyelin synthase | Down | MSTRG.10992.1; MSTRG.4667.1; MSTRG.4667.2; MSTRG.4667.3; MSTRG.4667.4; MSTRG.4667.5 | ko00600, ko04071 |
| CYC | cytochrome c | Down | MSTRG.20790.1 | ko01524, ko04115, ko04210, ko04214, ko04215, ko04932, ko05010, ko05012, ko05014, ko05016, ko05130, ko05131, ko05132, ko05134, ko05145, ko05152, ko05160, ko05161, ko05162, ko05163, ko05164, ko05167, ko05168, ko05169, ko05170, ko05200, ko05210, ko05222, ko05416 |
| CYP2J | cytochrome P450 family 2 subfamily J | Down | MSTRG.1674.2 | ko00590, ko00591, ko04726, ko04750, ko04913 |
| ACADL | long-chain-acyl-CoA dehydrogenase | Down | MSTRG.24102.4; MSTRG.24102.5; MSTRG.24102.6; MSTRG.24102.7; MSTRG.24102.8; MSTRG.24102.9 | ko00071, ko03320 |
| GCDH | glutaryl-CoA dehydrogenase | Down | MSTRG.15163.1 | ko00071, ko00310, ko00380 |
| IARS | isoleucyl-tRNA synthetase | Up | MSTRG.12564.1; MSTRG.12564.4; MSTRG.12564.7; MSTRG.12570.1 | ko00970 |
| LYSK | lysyl-tRNA synthetase, class I | Up | MSTRG.12117.1; MSTRG.12117.2; MSTRG.12118.1 | ko00970 |
| PARS | prolyl-tRNA synthetase | Up | MSTRG.4119.1 | ko00860, ko00970 |
| SEC13 | protein transport protein SEC13 | Up | MSTRG.10054.1 | ko04141 |
| SEC23 | protein transport protein SEC23 | Up | MSTRG.18846.10; MSTRG.18846.7; MSTRG.18846.9; MSTRG.18940.1; MSTRG.18940.8 | ko04141, ko05130 |
| UBQLN, DSK2 | Ubiquilin | Up | MSTRG.8956.1 | ko04141 |
| GLMS, GFPT | glutamine---fructose-6-phosphate transaminase (isomerizing) | Up | MSTRG.4125.10; MSTRG.4125.12; MSTRG.4125.2; MSTRG.4125.3; MSTRG.4125.4; MSTRG.4125.5; MSTRG.4126.4 | ko00250, ko00520, ko04931 |
| SLC15A1, PEPT1 | solute carrier family 15 (oligopeptide transporter), member 1 | Up | MSTRG.23029.2; MSTRG.23029.3; MSTRG.23029.5; MSTRG.23029.6; MSTRG.23029.7 | ko04974 |
