# Supplementary material for: Combined transcriptome and metabolome analysis reveal key regulatory genes and pathways of feed conversion efficiency of oriental river prawn Macrobrachium nipponense
Source: BMC Genomics. 2023 May 19;24:267. doi: 10.1186/s12864-023-09317-1 (PMC10197838; doi:10.1186/s12864-023-09317-1)
Supplement: Supplementary file 3 — Additional file 3: Table S3. Description of the DEGs of HRFI and LRFI groupsin muscles. [file 12864_2023_9317_MOESM3_ESM.docx]

| **Table S3 Description of the DEGs of HRFI and LRFI groups in muscles.** | | | | |
| --- | --- | --- | --- | --- |
| **Gene Symbol** | **Description** | **Regulation** | **Gene Id** | **KEGG Pathway** |
| GLB1, ELNR1 | beta-galactosidase | Down | MSTRG.21305.1; MSTRG.313.2 | ko00052, ko00511, ko00531, ko00600, ko00604, ko04142 |
| CTSB | cathepsin B | Down | MSTRG.15774.2; MSTRG.15774.4; MSTRG.15775.1; MSTRG.15775.7; MSTRG.17068.1 | ko04140, ko04142, ko04210, ko04612, ko04621, ko04924 |
| PRSS1_2_3 | Trypsin | Down | MSTRG.6354.10; MSTRG.6354.4; MSTRG.6354.7 | ko04080, ko04972, ko04974, ko05164 |
| CPA1 | carboxypeptidase A1 | Down | MSTRG.3313.1 | ko04972, ko04974 |
| PGM | Phosphoglucomutase | Down | MSTRG.23182.1; MSTRG.23183.1; MSTRG.23875.1; MSTRG.23883.1 | ko00010, ko00030, ko00052, ko00230, ko00500, ko00520 |
| HK | Hexokinase | Down | MSTRG.3957.1 | ko00010, ko00051, ko00052, ko00500, ko00520, ko00524, ko04066, ko04910, ko04930, ko04973, ko05131, ko05230 |
| GPI | glucose-6-phosphate isomerase | Down | MSTRG.14126.6 | ko00010, ko00030, ko00500, ko00520 |
| PFK9 | 6-phosphofructokinase | Down | MSTRG.26682.3 | ko00010, ko00030, ko00051, ko00052, ko03018, ko04066, ko04152, ko04922, ko05230 |
| PK, PYk | pyruvate kinase | Down | MSTRG.5090.1 | ko00010, ko00230, ko00620, ko04922, ko04930, ko05165, ko05203, ko05230 |
| LDH | L-lactate dehydrogenase | Down | MSTRG.16903.2 | ko00010, ko00270, ko00620, ko00640, ko04066, ko04922, ko05230 |
| ADH1_7 | alcohol dehydrogenase 1/7 | Down | MSTRG.14541.2; MSTRG.14541.4; MSTRG.16241.1 | ko00010, ko00071, ko00350, ko00830, ko00980, ko00982, ko05204 |
| AMY, AMYA, MALS | alpha-amylase | Down | MSTRG.2187.1; MSTRG.22498.3; MSTRG.22498.4; MSTRG.22498.5; MSTRG.980.1 | ko00500, ko04972, ko04973 |
| CYP1A1 | cytochrome P450 family 1 subfamily A1 | Down | MSTRG.1243.1 | ko00830, ko04726, ko05204 |
| RDH8 | retinol dehydrogenase 8 | Down | MSTRG.15128.2; MSTRG.15128.3 | ko00830 |
| CYP2A6 | cytochrome P450 family 2 subfamily A6 | Down | MSTRG.1243.1 | ko00830, ko04726, ko05204 |
| PNLIPRP1, PLRP1 | pancreatic lipase-related protein 1 | Down | MSTRG.4127.1 | ko00561, ko04972, ko04975 |
| CCNA | cyclin-A | Up | MSTRG.4507.1 | ko04110, ko04152, ko04218, ko04914, ko05161, ko05165, ko05166, ko05169, ko05200, ko05202, ko05203, ko05221 |
| CCNH | cyclin H | Up | MSTRG.24167.1 | ko03022, ko03420, ko04110 |
| ATP1A | sodium/potassium-transporting ATPase subunit alpha | Up | MSTRG.14075.1; MSTRG.14075.2; MSTRG.14076.1; MSTRG.8590.1 | ko04022, ko04024, ko04260, ko04261, ko04911, ko04918, ko04919, ko04925, ko04960, ko04961, ko04964, ko04970, ko04971, ko04972, ko04973, ko04974, ko04976, ko04978 |
| POLA1 | DNA polymerase alpha subunit A | Up | MSTRG.14983.1 | ko03030 |
| POLA2 | DNA polymerase alpha subunit B | Up | MSTRG.11954.1 | ko03030 |
| GYS | glycogen synthase | Up | MSTRG.6373.1 | ko00500, ko04151, ko04152, ko04910, ko04922, ko04931 |
| GGT | gamma-glutamyltranspeptidase / glutathione hydrolase | Up | MSTRG.20975.1; MSTRG.20977.1 | ko00430, ko00480 |
| IDH1, IDH2, ICD | isocitrate dehydrogenase | Up | MSTRG.11962.3; MSTRG.11962.5 | ko00020, ko00480, ko04146 |
| TUBA | tubulin alpha | Up | MSTRG.1004.1; MSTRG.24988.1; MSTRG.24989.2; MSTRG.8484.1; MSTRG.8485.1; MSTRG.9898.1; MSTRG.9899.1 | ko04145, ko04210, ko04530, ko04540, ko05010, ko05016, ko05130 |
