# Supplementary material for: Combined transcriptome and metabolome analysis reveal key regulatory genes and pathways of feed conversion efficiency of oriental river prawn Macrobrachium nipponense
Source: BMC Genomics. 2023 May 19;24:267. doi: 10.1186/s12864-023-09317-1 (PMC10197838; doi:10.1186/s12864-023-09317-1)
Supplement: Supplementary file 4 — Additional file 4: Table S4. Description of DEGs shared by the twoexperimental groups. [file 12864_2023_9317_MOESM4_ESM.docx]

| **Table S4 Description of DEGs shared by the two experimental groups.** | | | | |
| --- | --- | --- | --- | --- |
| **Gene Symbol** | **Description** | **Regulation** | **Gene Id** | **KEGG Pathway** |
| CTSL | cathepsin L, partial | Down | MSTRG.4372.6 | ko04142 |
| CHIB | chitinase 1B | Down | MSTRG.19644.1 | ko00520 |
| CHIA | chitinase 3A | Down | MSTRG.5616.1 | ko00520 |
| ND1 | NADH dehydrogenase subunit 1 | Down | MSTRG.1701.2； MSTRG.1702.21； MSTRG.1702.23； MSTRG.1702.25； MSTRG.1702.27； MSTRG.1702.31 | ko00190, ko04714, ko04723, ko05010, ko05012, ko05016 |
| ADH1_7 | alcohol dehydrogenase 1/7 | Down | MSTRG.14541.4 | ko00010, ko00071, ko00350, ko00830, ko00980, ko00982, ko05204 |
| CYP2A6 | cytochrome P450 family 2 subfamily A6 | Down | MSTRG.1243.1 | ko00830, ko04726, ko05204 |
| GFPT2 | Glucosamine-fructose-6-phosphate aminotransferase isomerising | Up | MSTRG.4125.2； MSTRG.4125.5 | ko00250, ko00520, ko04931 |
| UBA1 | ubiquitin-like modifier-activating enzyme 1 isoform X4 | Up | MSTRG.8658.8 | ko04120, ko05012 |
| SEC24C | protein transport protein Sec24C-like | Up | MSTRG.18846.7； MSTRG.18846.9 | ko04141, ko05130 |
