# Supplementary material for: Combined transcriptome and metabolome analysis reveal key regulatory genes and pathways of feed conversion efficiency of oriental river prawn Macrobrachium nipponense
Source: BMC Genomics. 2023 May 19;24:267. doi: 10.1186/s12864-023-09317-1 (PMC10197838; doi:10.1186/s12864-023-09317-1)
Supplement: Supplementary file 5 — Additional file 5: Table S5. Description of DEMs of the HRFI and LRFI groupsin hepatopancreas. [file 12864_2023_9317_MOESM5_ESM.docx]

| **Table S5 Description of DEMs of the HRFI and LRFI groups in hepatopancreas.** | | | | |
| --- | --- | --- | --- | --- |
| **Metabolites** | **Regulation** | **log2(FC)** | **P-value** | **VIP** |
| Valyl-Proline | Down | -2.6690 | 0.0066 | 4.8908 |
| Leucylproline | Down | -2.5618 | 0.0063 | 4.6334 |
| 1,4-beta-D-Glucan | Down | -2.0573 | 3.83E-06 | 3.2542 |
| Tyrosyl-Valine | Down | -1.8516 | 0.0069 | 3.4862 |
| Isoleucyl-Aspartate | Down | -1.2075 | 0.0165 | 3.3653 |
| Actinonin | Down | -1.1530 | 0.0037 | 1.0842 |
| Glutamylleucine | Down | -1.0028 | 0.0107 | 7.2880 |
| Butenylcarnitine | Down | -0.9984 | 0.0003 | 1.4603 |
| Propenoylcarnitine | Down | -0.9859 | 0.0008 | 1.5408 |
| Allopurinol-1-ribonucleoside | Down | -0.8895 | 0.0019 | 3.4287 |
| Valyl-Leucine | Down | -0.7659 | 0.0079 | 3.9843 |
| (2E,4Z)-decadienoylcarnitine | Down | -0.7592 | 0.0129 | 1.0599 |
| Alanyl-Isoleucine | Down | -0.7381 | 0.0242 | 3.3354 |
| Valyl-Gamma-glutamate | Down | -0.7370 | 0.0028 | 4.5194 |
| Valyl-Glutamate | Down | -0.7094 | 0.0048 | 3.7494 |
| Alanyl-dl-Leucine | Down | -0.6382 | 0.0190 | 5.1959 |
| Guanine | Down | -0.6177 | 0.0230 | 1.5180 |
| Valyl-Threonine | Down | -0.6092 | 0.0145 | 3.2439 |
| Isoleucyl-Glutamate | Down | -0.4888 | 0.0407 | 4.0508 |
| Aspartyl-Leucine | Down | -0.4330 | 0.0299 | 3.7520 |
| Isoleucyl-Threonine | Down | -0.4274 | 0.0473 | 3.4001 |
| L-Arginine | Up | 0.4713 | 0.023 | 6.3143 |
| L-Isoleucine | Up | 0.5481 | 0.0444 | 13.6515 |
| L-Lysine | Up | 0.5945 | 0.0175 | 5.4373 |
| L-Proline | Up | 0.6406 | 0.0213 | 6.6474 |
| O-propanoyl-D-carnitine | Up | 0.9375 | 0.0191 | 5.6814 |
| Betaine | Up | 1.0770 | 0.0386 | 11.7904 |
| Cytosine | Up | 1.1221 | 8.56E-08 | 1.9489 |
| Xanthine | Up | 1.4202 | 0.0002 | 3.8689 |
| Cassythine | Up | 1.4650 | 0.0024 | 1.5467 |
| LMFA07070066 (Glutarylcarnitine) | Up | 1.6707 | 0.0091 | 1.6353 |
| Aspirin | Up | 2.5583 | 0.0023 | 2.4544 |

**Note:** VIP variable weight value: The VIP value from the OPLS-DA model. The larger the VIP, the greater the contribution of the variable to the grouping.
