# Supplementary material for: Combined transcriptome and metabolome analysis reveal key regulatory genes and pathways of feed conversion efficiency of oriental river prawn Macrobrachium nipponense
Source: BMC Genomics. 2023 May 19;24:267. doi: 10.1186/s12864-023-09317-1 (PMC10197838; doi:10.1186/s12864-023-09317-1)
Supplement: Supplementary file 6 — Additional file 6: Table S6. Description of DEMs of HRFI and LRFI groups inmuscles. [file 12864_2023_9317_MOESM6_ESM.docx]

| **Table S6 Description of DEMs of HRFI and LRFI groups in muscles.** | | | | |
| --- | --- | --- | --- | --- |
| **Metabolites** | **Regulation** | **log2(FC)** | **P-value** | **VIP** |
| Glycyl-Glutamate | Down | -1.9794 | 0.0021 | 1.6351 |
| Uridine | Down | -1.2666 | 0.0034 | 1.5356 |
| D-Glucose 6-phosphate | Down | -1.2530 | 0.0496 | 5.7266 |
| Glutathionate(1-) | Down | -0.6799 | 0.0226 | 1.6095 |
| Adenosine monophosphate | Down | -0.5230 | 0.0358 | 6.7175 |
| L-Arginine | Up | 0.2205 | 0.0019 | 19.2481 |
| trans-Hexadec-2-enoyl carnitine | Up | 0.3454 | 0.0034 | 1.4329 |
| Hexacosanoyl carnitine | Up | 0.3689 | 0.0027 | 1.6628 |
| Tricosanoylglycine | Up | 0.3797 | 0.0095 | 2.6314 |
| L-Histidine | Up | 0.3954 | 0.0318 | 5.8995 |
| Choline | Up | 0.4657 | 0.0054 | 6.3509 |
| Allopurinol-1-ribonucleoside | Up | 0.5347 | 0.0080 | 9.3532 |
| Uracil | Up | 0.5814 | 0.0001 | 2.8405 |
| Salicylaldehyde | Up | 0.6321 | 0.0137 | 1.8408 |
| Allopurinol | Up | 0.6583 | 0.0002 | 7.9840 |
| 1,4-beta-D-Glucan | Up | 0.6743 | 0.0003 | 12.0391 |
| 2-Hydroxycinnamic acid | Up | 0.7013 | 0.0301 | 8.6466 |
| Propionylcarnitine | Up | 0.7237 | 0.0010 | 3.9606 |
| L-Acetylcarnitine | Up | 0.8832 | 0.0018 | 9.9998 |
| Tyramine | Up | 0.9343 | 0.0332 | 5.7955 |
| Valyl-Lysine | Up | 0.9365 | 0.0266 | 2.0668 |
| Acetyl-D-carnitine | Up | 1.0329 | 0.0003 | 7.4715 |
| L-Lysine | Up | 1.0440 | 0.0112 | 6.2522 |
| Adenylsuccinic acid | Up | 1.0552 | 0.0023 | 6.3489 |
| 4-heptynoic acid | Up | 1.0641 | 1.42E-05 | 13.266 |
| Arachidyl carnitine | Up | 1.1142 | 0.0397 | 1.5497 |
| D-Glutamine | Up | 1.1237 | 0.0009 | 3.3879 |
| Valproylglycine | Up | 1.1945 | 0.0080 | 3.8279 |
| Nicotinamide adenine dinucleotide (NAD) | Up | 1.2202 | 0.0041 | 4.3854 |
| L-Glutamine | Up | 1.2291 | 0.0050 | 4.2091 |
| L-Glutamate | Up | 1.2666 | 0.0194 | 5.1418 |
| Retinylphosphate mannose | Up | 1.3246 | 0.0221 | 2.3650 |
| Hypoxanthine | Up | 1.3726 | 0.0007 | 1.1924 |
| Phosphocholine | Up | 1.6164 | 0.0219 | 4.8661 |
| 3-Aminosalicylic acid | Up | 1.7564 | 1.11E-05 | 3.5602 |
| Argininosuccinic acid | Up | 1.9058 | 0.0233 | 4.1067 |
| L-Ornithine | Up | 2.0765 | 0.0223 | 2.8338 |
| Aspirin | Up | 2.0917 | 0.0279 | 1.2871 |

**Note:** VIP variable weight value: The VIP value from the OPLS-DA model. The larger the VIP, the greater the contribution of the variable to the grouping.
