# Supplementary material for: Combined transcriptome and metabolome analysis reveal key regulatory genes and pathways of feed conversion efficiency of oriental river prawn Macrobrachium nipponense
Source: BMC Genomics. 2023 May 19;24:267. doi: 10.1186/s12864-023-09317-1 (PMC10197838; doi:10.1186/s12864-023-09317-1)
Supplement: Supplementary file 7 — Additional file 7: Table S7. Comprehensive analysis of the metabolic enrichment pathways bymulti-omics. [file 12864_2023_9317_MOESM7_ESM.docx]

| **Table S**7 Comprehensive analysis of the metabolic enrichment pathways by multi-omics. | | | | | | |
| --- | --- | --- | --- | --- | --- | --- |
| Tissues | KEGG ID | Term | Transcriptome | | Metabolomic | |
|  |  |  | Genes | Regulation | Metabolites | Regulation |
| Hepatopancreas | ko00564 | Glycerophospholipid metabolism | glycerol-3-phosphate O-acyltransferase 1/2 | Down | Glycerophosphocholine; | Up |
|  |  |  | 1-acyl-sn-glycerol-3-phosphate acyltransferase | Down | Glycerylphosphorylethanolamine; | Up |
|  |  |  | diacylglycerol kinase (ATP) | Down | Acetylcholine; | Down |
|  |  |  | lysophosphatidic acid acyltransferase / lysophosphatidylinositol acyltransferase | Down |  |  |
|  |  |  | lysocardiolipin and lysophospholipid acyltransferase | Down |  |  |
|  |  |  | lysophosphatidylcholine acyltransferase / lyso-PAF acetyltransferase | Down |  |  |
|  |  |  | phosphatidylserine synthase 2 | Down |  |  |
|  |  |  | secretory phospholipase A2 | Down |  |  |
|  |  |  | glycerophosphocholine phosphodiesterase GPCPD1 | Down |  |  |
|  |  |  | diacylglycerol cholinephosphotransferase | Down |  |  |
|  |  |  | acetylcholinesterase | Down |  |  |
|  |  |  | ethanolaminephosphotransferase | Down |  |  |
|  |  |  | ethanolamine kinase | Down |  |  |
|  |  |  | ethanolamine-phosphate phospho-lyase | Down |  |  |
|  |  |  | cardiolipin synthase (CMP-forming) | Down |  |  |
|  |  |  | phospholipase D1/2 | Down |  |  |
|  |  |  | phosphatidate phosphatase | Down |  |  |
|  |  |  | choline-phosphate cytidylyltransferase | Up |  |  |
|  | ko00565 | Ether lipid metabolism | diacylglycerol cholinephosphotransferase | Down | Glycerophosphocholine; | Up |
|  |  |  | secretory phospholipase A2 | Down | Glycerylphosphorylethanolamine; | Up |
|  |  |  | Ethanolaminephosphotransferase | Down |  |  |
|  |  |  | phosphatidate phosphatase | Down |  |  |
|  |  |  | platelet-activating factor acetylhydrolase | Down |  |  |
|  |  |  | ectonucleotide pyrophosphatase/phosphodiesterase family member 6 | Down |  |  |
|  |  |  | phospholipase D1/2 | Down |  |  |
|  |  |  | galactosylceramide sulfotransferase | Up |  |  |
|  | ko00970 | Aminoacyl-tRNA biosynthesis | glutamyl-tRNA synthetase | Up | L-Lysine; | Up |
|  |  |  | glutaminyl-tRNA synthetase | Up | L-Arginine; | Up |
|  |  |  | alanyl-tRNA synthetase | Up | L-Histidine; | Up |
|  |  |  | asparaginyl-tRNA synthetase | Up | L-Proline; | Up |
|  |  |  | seryl-tRNA synthetase | Up | L-Isoleucine; | Up |
|  |  |  | isoleucyl-tRNA synthetase | Up |  |  |
|  |  |  | lysyl-tRNA synthetase, class I | Up |  |  |
|  |  |  | prolyl-tRNA synthetase | Up |  |  |
| Muscle | ko00480 | Glutathione metabolism | glutamate--cysteine ligase catalytic subunit | Up | L-Glutamate; | Up |
|  |  |  | aminopeptidase N | Up | L-Ornithine; | Up |
|  |  |  | gamma-glutamyltranspeptidase / glutathione hydrolase | Up | gamma-Glutamylcysteine; | Down |
|  |  |  | isocitrate dehydrogenase | Up |  |  |
|  |  |  | 6-phosphogluconate dehydrogenase | Up |  |  |
|  |  |  | glucose-6-phosphate 1-dehydrogenase | Up |  |  |
|  |  |  | pyrimidodiazepine synthase | Up |  |  |
|  |  |  | spermidine synthase | Up |  |  |
|  |  |  | ribonucleoside-diphosphate reductase subunit M1 | Up |  |  |
|  |  |  | glutathione peroxidase | Down |  |  |
|  |  |  | spermine synthase | Down |  |  |
|  |  |  | glutathione S-transferase | Down |  |  |
