# Supplementary figures and images for: Combined transcriptome and metabolome analysis reveal key regulatory genes and pathways of feed conversion efficiency of oriental river prawn Macrobrachium nipponense
Source: BMC Genomics. 2023 May 19;24:267. doi: 10.1186/s12864-023-09317-1 (PMC10197838; doi:10.1186/s12864-023-09317-1)

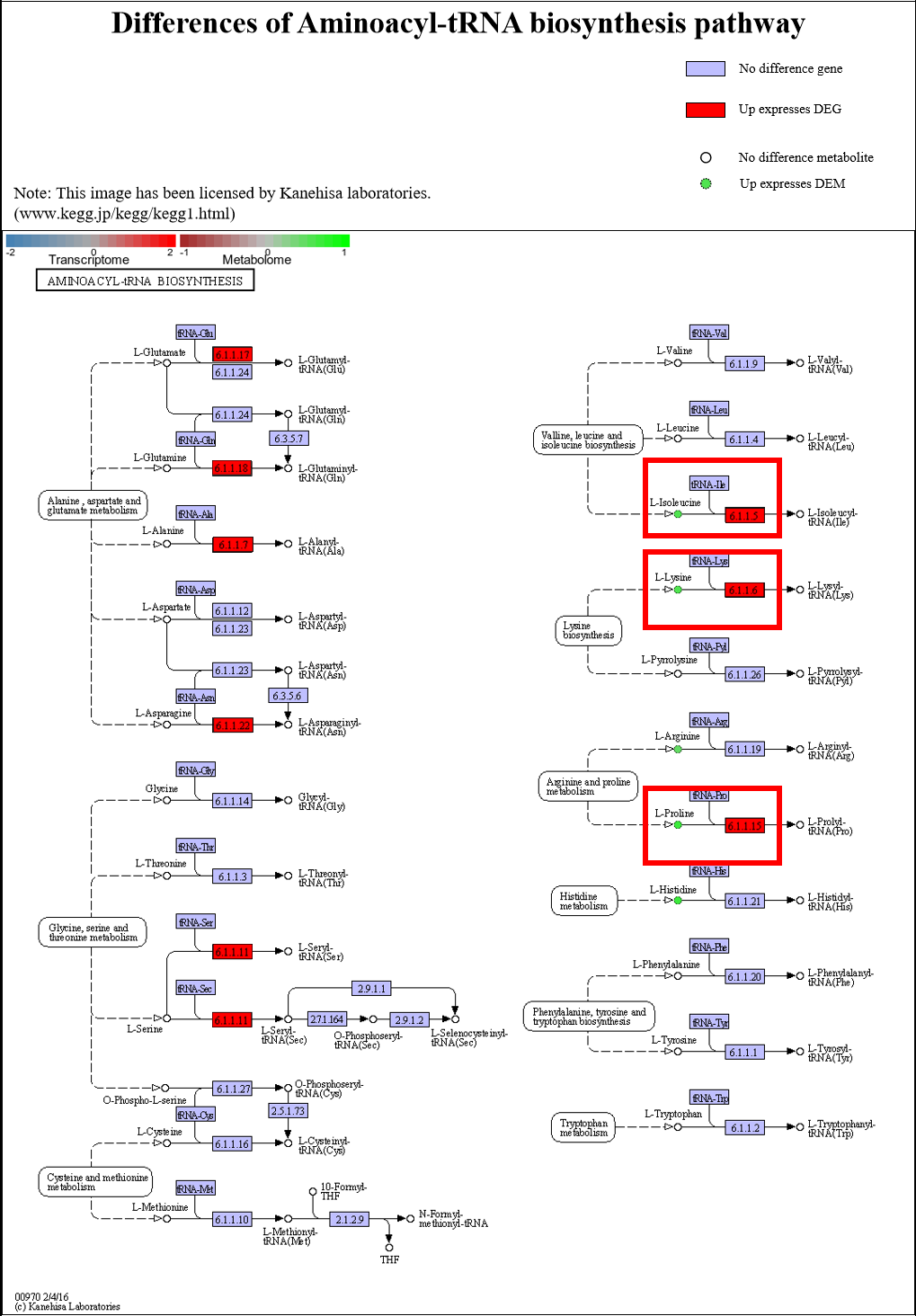

Supplement: Supplementary file 8 — Additional file 8: FigureS1. [file 12864_2023_9317_MOESM8_ESM.tif]

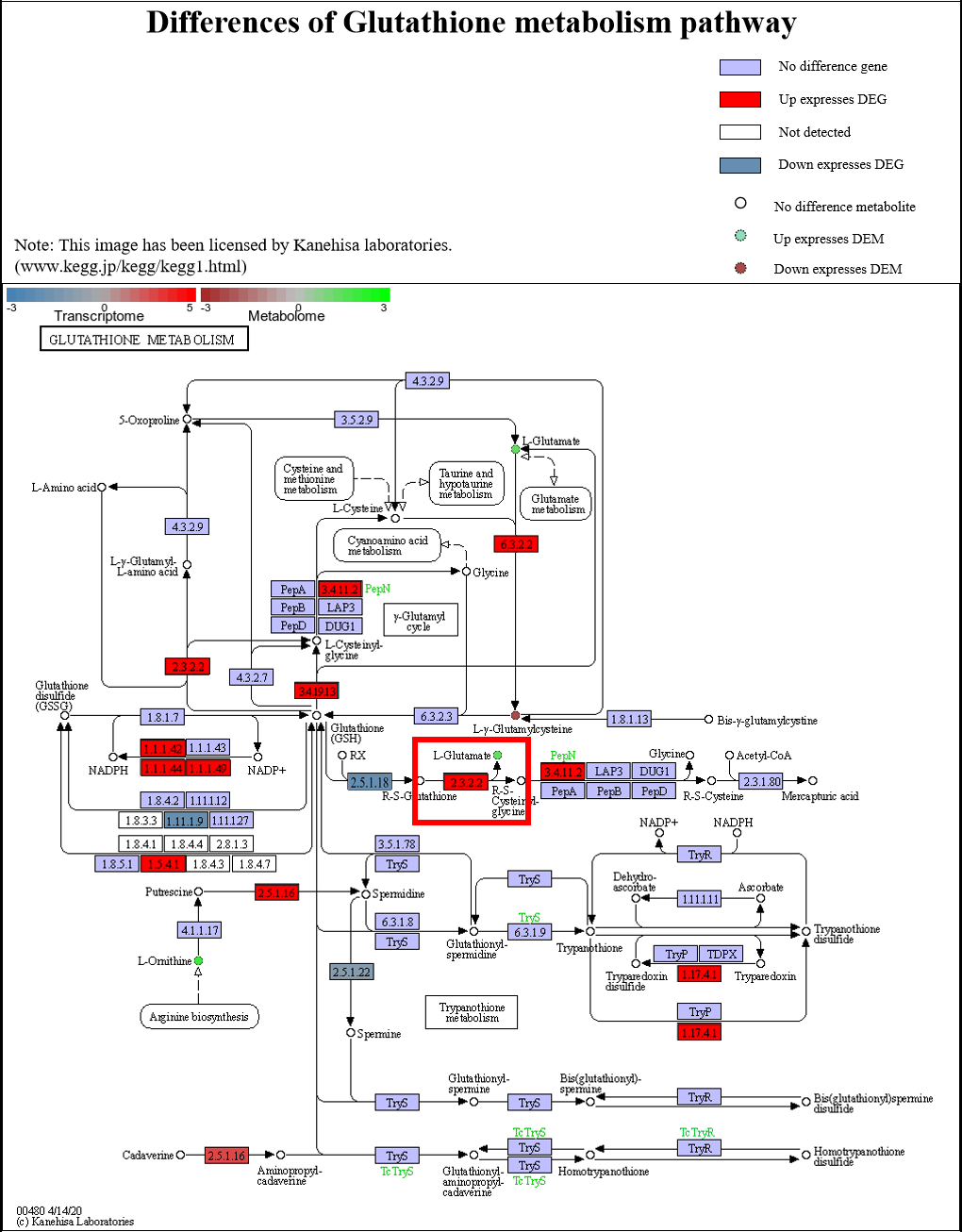

Supplement: Supplementary file 9 — Additional file 9: FigureS2. [file 12864_2023_9317_MOESM9_ESM.tif]
